# In silico analysis of potential inhibitors for breast cancer targeting 17beta‐hydroxysteroid dehydrogenase type 1 (17beta‐HSD1) catalyses

**DOI:** 10.1111/jcmm.18584

**Published:** 2024-08-12

**Authors:** Md. Rezaul Islam, Jehad Zuhair Tayyeb, Hridoy Kumar Paul, Mirza Nafeul Islam, Gbolahan Oladipupo Oduselu, Imren Bayıl, Magda H. Abdellattif, Khairia Mohammed Al‐Ahmary, Saedah R. Al‐Mhyawi, Magdi E. A. Zaki

**Affiliations:** ^1^ Department of Pharmacy, Faculty of Allied Health Sciences Daffodil International University Dhaka Bangladesh; ^2^ Department of Clinical Biochemistry, College of Medicine University of Jeddah Jeddah Saudi Arabia; ^3^ Department of Pharmacy Jashore University of Science and Technology Jashore Bangladesh; ^4^ Department of Pharmacy University of Rajshahi Rajshahi Bangladesh; ^5^ Covenant University Bioinformatics Research Ota Nigeria; ^6^ Department of bioinformatics and computational biology Gaziantep University Gaziantep Turkey; ^7^ Department of Chemistry, Sciences College University College of Taraba, Taif University Taif Saudi Arabia; ^8^ Department of Chemistry, College of Science University of Jeddah Jeddah Saudi Arabia; ^9^ Department of Chemistry, College of Science Imam Mohammad Ibn Saud Islamic University Riyadh Riyadh Saudi Arabia

**Keywords:** breast cancer, DFT calculation, in silico studies, molecular docking, molecular dynamics simulation, pass prediction, retinoic acid

## Abstract

Breast cancer (BC) is still one of the major issues in world health, especially for women, which necessitates innovative therapeutic strategies. In this study, we investigated the efficacy of retinoic acid derivatives as inhibitors of 17beta‐hydroxysteroid dehydrogenase type 1 (17beta‐HSD1), which plays a crucial role in the biosynthesis and metabolism of oestrogen and thereby influences the progression of BC and, the main objective of this investigation is to identify the possible drug candidate against BC through computational drug design approach including PASS prediction, molecular docking, ADMET profiling, molecular dynamics simulations (MD) and density functional theory (DFT) calculations. The result has reported that total eight derivatives with high binding affinity and promising pharmacokinetic properties among 115 derivatives. In particular, ligands 04 and 07 exhibited a higher binding affinity with values of −9.9 kcal/mol and −9.1 kcal/mol, respectively, than the standard drug epirubicin hydrochloride, which had a binding affinity of −8.2 kcal/mol. The stability of the ligand‐protein complexes was further confirmed by MD simulations over a 100‐ns trajectory, which included assessments of hydrogen bonds, root mean square deviation (RMSD), root mean square Fluctuation (RMSF), dynamic cross‐correlation matric (DCCM) and principal component analysis. The study emphasizes the need for experimental validation to confirm the therapeutic utility of these compounds. This study enhances the computational search for new BC drugs and establishes a solid foundation for subsequent experimental and clinical research.

## 1 INTRODUCTION

Among several types of cancer, one of the most common and potentially fatal cancers affecting women globally is breast cancer (BC), among many others. The worldwide burden of disease caused by the most frequent malignant tumour, BC, has emerged as a significant threat to global health, particularly women's health.^1^ According to the World Health Organization (WHO) (https://www.who.int/news‐room/fact‐sheets/detail/breast‐cancer; accessed April 10, 2024), the burden of BC varies considerably depending on human development. In countries with a remarkably high Human Development Index (HDI), 1 in 12 women will develop BC and 1 in 71 women will die from it, while in countries with a low HDI, 1 in 27 women will be diagnosed with BC but 1 in 48 women will die to the disease. Although BC is primarily a women's disease (around 99% of cases occur in women), it can also affect men, rarely (0.5%–1% of cases).^2^

Although a considerable number of people are affected by BC day by day, current therapeutic options are limited. However, 30% of patients with early‐stage BC get recurrent disease, mostly metastases. The therapy itself is associated with various side effects.^3^, ^4^ This highlights the critical need for novel strategic therapeutics specifically to the treatment of BC.

Retinoids constitute, a class of chemicals, and the active form of vitamin A, known as all‐trans‐retinoic acid, plays a significant role in controlling cell development and differentiation, regulating immunological competence, and influencing various physiological processes across multiple organ systems.^5^, ^6^ Some studies have reported that retinoic acid derivatives have shown promise in the fight against cancer by modulating vital cellular processes.^7^ It also regulates gene expression by binding to certain receptors; these receptors impact cell cycle progression, death, and differentiation.^8^, ^9^ So, in this investigation, retinoic acid and its derivatives are selected as promising therapeutic targets due to their number of pharmacological actions.

Besides, earlier research has investigated retinoic acid for its significant impact on embryonic development and tissue regeneration. Studies have shown that it can induce stem cell differentiation into specific cell lineages, promising the development of novel therapies for degenerative diseases and tissue injuries.^10^ Additionally, retinoic acid derivatives have demonstrated potent anticancer properties, showing their ability to inhibit cancer cell proliferation and induce differentiation, thereby slowing tumour growth.^11^, ^12^, ^13^, ^14^ Furthermore, retinoic acid derivatives have been linked to the maintenance and function of the immune system. Research has demonstrated their role in improving the body's defences against infections and autoimmune disorders and their ability to regulate immune responses.^15^ Based on the literature study, we selected retinoic acid derivatives to identify potential drug candidates against BC.

17beta‐HSD1 has focused on this investigation as a target receptor for the development of anti‐BC drug discovery since this enzyme (17beta‐HSD1) leads the biosynthesis and metabolism of oestrogen plays a significant role to the proliferation and progression of the BC. Targeting 17beta‐HSD1 might be promising receptor to the development of anti‐cancer drugs that could lead to more precise and efficient treatments of BC.^16^, ^17^, ^18^ Inhibition of 17beta‐HSD1 can reduce oestrogen levels and prevent the progression of BC. Recent study found that a covalent inhibitor (called PBRM) targeting the enzyme (17beta‐HSD1) showed the ability to block the pathway of oestrogen biosynthesis and reduce tumour growth in BC models, with no toxic effects observed.^19^

We applied computational drug design approaches to determine the structural details of the retinoic acid derivatives and how they interact with biological targets involved in the pathogenesis of BC. Because the traditional method of drug development and drug discovery process requires years of testing and substantial financial expenditure are often required. However, this conventional procedure can be minimized with computational techniques since they speed up the process of finding and optimising possible medication candidates.^20^, ^21^, ^22^

## 2 MATERIALS AND METHODS

### 2.1 PASS prediction

The biological activity of retinoic acid derivatives can be predicted with the help of the PASS online tools. We obtained information on a wide range of biological actions, including antibacterial, antiviral, and antineoplastic effects, by uploading the Isomeric SMILES to the PASS online website (http://way2drug.com/).^23^ With the help of this web tool, which has over 4,000 features covering both drug and non‐drug activities, possible bioactive compounds can be identified with 90% accuracy.^24^, ^25^ The terms Pa (probability for active molecule) and Pi (probability for inactive molecule) describe the values that the PASS online web provides. For a molecule to be classified as potential, its Pa and Pi values need to be between 0.00 and 1.00, with Pa + Pi ≠ 1.^26^, ^27^

### 2.2 Preparation of ligand, and DFT calculation

For optimization, retinoic acid derivatives SDF‐formatted structure files were obtained from the PubChem database.^28^ They transferred to GaussView 09 software as an MDL mol file, all the 3D chemical structures of the studied molecules.^29^

The theoretical calculations were performed by Gaussian 09 in the gas phase using the DFT method/ 6‐311G (d,p) basis set with B3LYP functional, to obtain the most stable conformation to rearrange the atomic components and the geometrical arrangement of the molecules. Gauss View 05 was used to enhance the visualization of the results.^30^, ^31^ This is necessary since ligands with the ideal geometrical arrangement are needed for molecular docking and molecular dynamics (MD) simulation. The optimised chemical structures were preserved in PDB format.

### 2.3 Protein preparation and molecular docking study

The three‐dimensional (3D) structures of 17beta‐HSD type 1, associated with BC (PDB ID: 3HB5), were selected and downloaded from the Protein Data Bank.^16^ Subsequently, using BIOVIA Discovery Studio 2021, heteroatoms were removed, and the protein was prepared and saved in PDB format.

To determine the binding affinities of the ligand and protein, we employed the PyRx software to perform molecular docking. Proteins and ligands were both loaded to the PyRx application where protein considered as macromolecules in molecular docking. Maximal energy and grid surface area such as grid's centre and dimensions were used to molecular docking. During molecular docking, mentioned grid box with coordinates were used X = −29.55, Y = −33.986, and Z = 26.458 as a grid centre, and the dimensioned was at X = −43.653, Y = −22.352, and Z = −42.6601. After the completion of the docking process, the binding affinity of the compounds was determined by analysing their interactions with the target protein. Discovery Studio, Pymol and Chimera application were employed for further analysis and visualisation of these complexes.

### 2.4 Determination of ADMET profile and pharmacokinetics

To prevent failure during the clinical trial studies, the ADMET properties—absorption, distribution, metabolism, excretion and toxicity—are critical to the drug development process,^32^ and we therefore evaluated the pharmacokinetic properties of all compounds in silico. Utilising the pkCSM online server (http://biosig.unimelb.edu.au/pkcsm/prediction), we estimated the pharmacokinetic properties of these substances.^33^ The pharmacokinetic profile of selected compounds is investigated in this online database with respect to water solubility, toxicity, metabolism, distribution and absorption.

### 2.5 Molecular dynamics simulation (MDs)

MD simulations play a significant role in advancing drug development research by providing crucial insights into the dynamic behaviour and stability of protein‐ligand complexes.^34^ To explore potential interactions, a 100‐ns MD simulation was initiated for ligand 04, ligand 07, and standard with the BC protein (PDB ID: 3HB5). The simulations employed the CHARMM36 force field^35^ and the GROMACS version 2023 software package.^36^ To generate a suitable molecular topology file for the CHARMM36 force field, the SwissParam online platform was utilized,^37^ and an explicit water model was employed. To neutralize the charge of the system, sodium ions (Na+) and chloride ions (Cl–) were added accordingly.

The MD simulations involved structure minimisation using the CHARMM36 force field, equilibration at 310 K for 100 seconds in the NPT ensemble, and constraint of hydrogen atoms using the Lincs technique. Van der Waals forces were calculated with a cut‐off value using a switching method of 12–14, and long‐range electrostatic interactions were computed using the particle mesh Ewald (PME) approach.^38^ The simulations maintained a temperature of 310 K, and the system size adjustments for the barostat were targeted at 1 bar. An integration time step of 2 fs and a production run of 50,000,000 steps resulted in a total simulation time of 100 ns. Following the MD simulations, trajectory data were analysed and visualized using VMD 1.9.3, R studio 4.3.1 Bio3d versions on the Galaxy Europe platform,^39^ and XMGRACE (version 5.1.19). Various characteristics, including root mean square deviation (RMSD), root mean square fluctuation (RMSF), hydrogen bonds (H‐bonds), principal component analysis (PCA), and dynamics cross‐correlation map (DCCM), were assessed to gain insights into the behaviour of the ligand 04‐3HB5, ligand 07‐3HB5, and Epirubicin hydrochloride‐3HB5 complexes.

## 3 RESULTS

### 3.1 PASS prediction

The potential bioactive compounds' probabilities of being active (Pa) and inactive (Pi) are evaluated by the pass prediction parameter. Pa and Pi have values between 0.00 and 1.0, and Pa + Pi ≠ 1 indicate that a compound cannot be completely active and inactive simultaneously.^40^, ^41^ In this investigation, total of 115 derivatives were initially taken, and their Pa and Pi score were determined against antineoplastic, antibacterial, and antiviral (Influenza) (Table S1). Afterward, it was reported that the Pa score for antineoplastic activity exceeds that for antibacterial and antiviral (Influenza). Among the 115 derivatives, those with PubChem CID: 6438629, 20849244, 5364256, 10253806, 71316382, 10427626, 14443943 and 101278260 exhibited the highest Pa values (Pa = 0.911–0.930) (Table 1), suggesting that they may possess greater biological activity than other derivatives, rendering them effective against neoplastic diseases such as cancer. Based on this prediction data, BC target protein has been selected for further investigation.

**TABLE 1:** Pass prediction spectrum of reported ligands.

No.	CID	Compounds name	Antineoplastic		Antibacterial		Antiviral (Influenza)	
			Pa	Pi	Pa	Pi	Pa	Pi
01	6438629	4‐Hydroxyretinoic acid	0.911	0.005	0.395	0.031	0.323	0.076
02	20849244	9‐cis‐4‐Hydroxyretinoic acid	0.911	0.005	0.395	0.031	0.323	0.076
03	5364256	5,8‐Monoepoxyretinoic acid	0.930	0.005	0.430	0.024	0.218	0.171
04	10253806	Retinoic acid glucose ester	0.912	0.005	0.581	0.010	0.557	0.016
05	71316382	5,8‐Epoxy‐13‐cis‐retinoic acid	0.930	0.005	0.430	0.024	0.218	0.171
06	10427626	4‐Methoxy retinoic acid methyl ester	0.923	0.005	0.329	0.050	0.248	0.134
07	14443943	5,8‐Epoxytretinoin	0.930	0.005	0.430	0.024	0.218	0.171
08	101278260	5,8‐Epoxy‐9‐cis retinoic acid	0.930	0.005	0.430	0.024	0.218	0.171

### 3.2 Molecular docking analysis against targeted receptor

Molecular docking was used to identify and visualize binding configurations and binding affinity between a hypothetical receptor with a ligand.^42^ The PyRx application in AutoDock vina function was utilized to assess potential binding affinities and interactions with the target protein of 17beta‐hydroxysteroid dehydrogenase type 1 (17beta‐HSD1) catalyses (PDB ID: 3HB5).^43^

Table 2 summarizes the predicted binding energies of selected retinoic acid derivatives to proteins involved in BC. The binding affinity ranges of the derivatives against the target receptor were reported to be between −8.00 kcal/mol and –9.9 kcal/mol, with the maximum affinity documented for ligands 04 and 07. In comparison, the standard Epirubicin hydrochloride exhibited a binding affinity of −8.2 kcal/mol against the target protein. When compared with our ligands, six of them demonstrated affinities greater than the standard.

**TABLE 2:** Binding affinities of docked ligand calculated against breast cancer targeted proteins.

No	PubChem CID	17beta‐hydroxysteroid dehydrogenase type 1 (17beta‐HSD1) catalyses (PDB ID: 3HB5)
		kcal/mol
01	6438629	−8.4
02	20849244	−8.0
03	5364256	−9.0
04	10253806	−9.9
05	71316382	−8.1
06	10427626	−8.7
07	14443943	−9.1
08	101278260	−8.8
09	Epirubicin hydrochloride	−8.2

### 3.3 Molecular docking and interaction analysis

**FIGURE 1:**
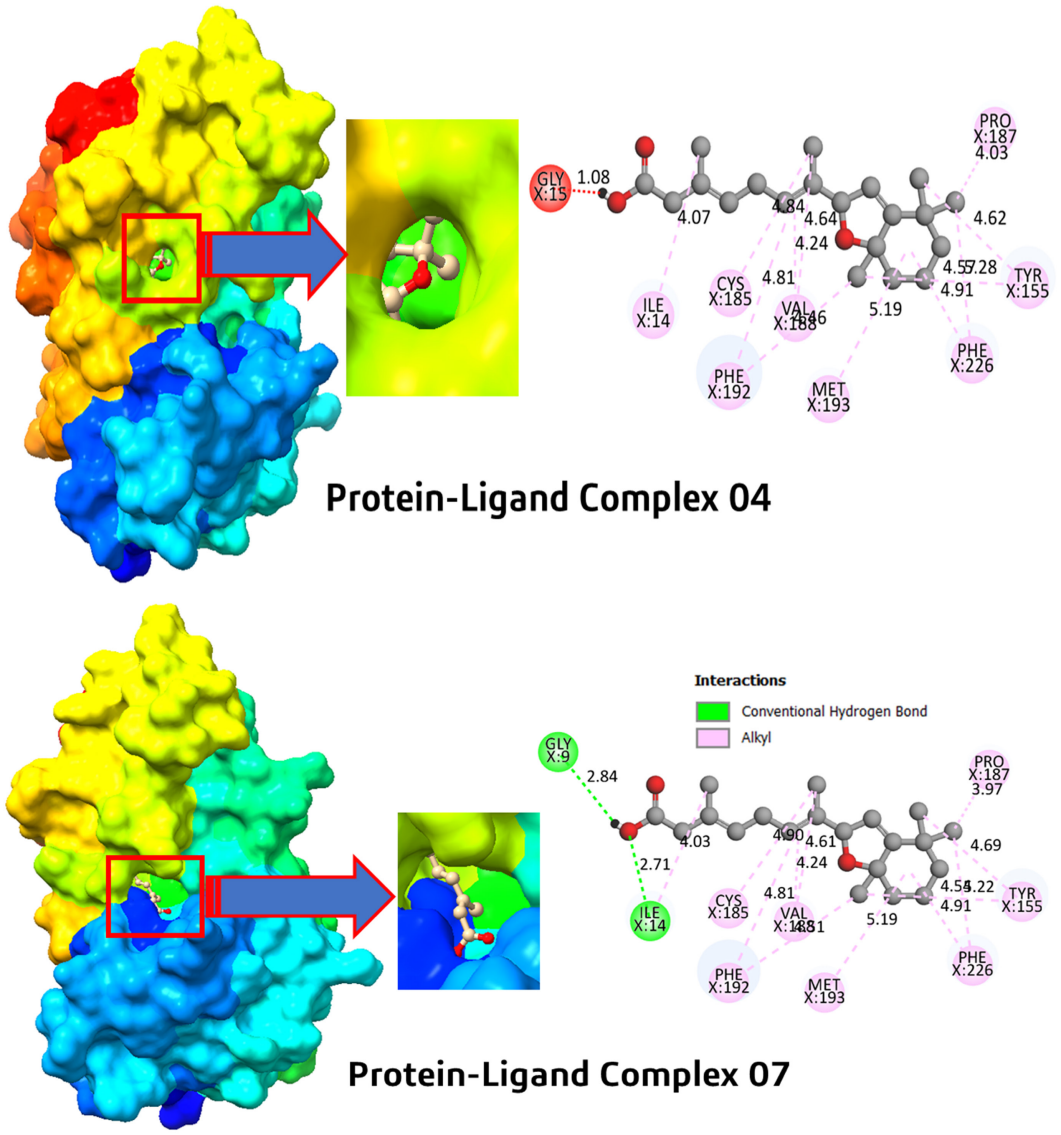
Illustration docking interaction of Protein‐Ligand complex 04, and 07. There are two distinct types of bonds are present mostly. The green colour is represented the conventional hydrogen bonds, and the pink colour described the alkyl bonds. However, one unfavourable bond is also seen, which illustrated by red colour.

Molecular docking and interaction techniques were employed to better understand the complex binding mechanisms between drugs and proteins, introducing the formation of active amino acid residues within the drug‐ 17beta‐HSD1 protein complexes. Figure 1 provides a visual depiction of the docking pocket, demonstrating the 2D interaction between the ligand and 17beta‐HSD1protein, along with the specific interactions of the ligand with protein residues. Using Pymol, Discovery Studio Visualizer and Chimera application.^44^, ^45^, ^46^ These three applications have different function such as the Pymol was used to make the ligand protein complex after molecular docking, discovery studio was used to visualize the second interaction of ligand‐protein complex, and the chimera is used to create, and visualize binding pocket region of the ligand protein.

Through investigating the three‐dimensional structure of active amino acid residues, we identified the different bond that formed during molecular docking. After that, it is seen that two types of bonds are formed in drug protein complex including conventional hydrogen bonds, and pi‐alkyl bonds (shown in green, and pink colour).

Hydrogen bonds are crucial due to their stability and specificity, influencing molecular recognition and binding affinity, where the hydrophobic bonds (alkyl) enhance the structural stability and binding force of the complexes.^47^, ^48^, ^49^

In the protein‐ligand complex 4 (glucose ester of retinoic acid) and 7 (5,8‐epoxyretinoin) in particular, several common alkyl bonds were formed with amino acid residues, including proline at position 187 (PRO187), cysteine at position 185 (CYS185), phenylalanine at positions 192 (PHE192) and 226 (PHE226), valine at position 188 (VAL188), methionine at position 193 (MET193) and tyrosine at position 155 (TYR155). The complexes differ in three different bindings: Protein‐ligand complex 4 has an unfavourable bond with glycine at position 15 (GLY15) and an alkyl bond with isoleucine at position 14 (ILE14) and contains no conventional hydrogen bonds, whereas protein‐ligand complex 7 has two conventional hydrogen bonds with glycine at position 9 (GLY9) and with isoleucine at position 14 (ILE14). The hydrogen bonds in complex 7 contribute to the specificity and stability of molecular recognition, along with alkyl bonds like those in complex 4. These results highlight the binding affinity of the ligands and suggest possible efficacy through molecular interactions at the active site.

### 3.4 ADME, and toxicity profile investigation

Any drug substances with inadequate ADME and toxicity properties can have adverse effects on the physiologic system, including organ impairment, hypersensitivity reactions, dermatologic irritation, and other adverse effects that sometimes cause life‐threatening problems. To avoid this complexity, the main ADMET properties of retinoic acid derivatives have been calculated and analysed.^50^ The pharmacokinetic ADMET properties of retinoic acid derivatives, which include solubility in water (LogS), human intestinal absorption (%), VDss (log L/kg), BBB permeability, CYP450 1A2 inhibitor and CYP450 2D6 substrate, total clearance (ml/min/kg), renal substrate for OCT2, AMES toxicity and skin sensitisation were investigated using the pkCSM method approach. All these properties influence the absorption, distribution, metabolism, and excretion of the drug molecules as well as their toxic effects on the body. A drug must exhibit these properties in an acceptable result in order to show adequate physiological activity and successfully reach the desired organ within the physiological system at a certain concentration.^51^

We have chosen the following two parameters to better understand the absorption aspect: human intestinal absorption, water solubility. The human intestinal absorption rate serves as a primary indicator of a compound's ability to be absorbed from the gastrointestinal tract into the bloodstream. Data gathered from the pkCSM server indicates that the human intestinal absorption rate of the Compound 04 had the lowest score of 66.66%, while all our suggested compounds have human intestinal absorption rates of greater than 90%.

Water solubility, quantified in Log S, plays a crucial role in determining the dissolution of a compound and subsequently its absorption. The investigation has shown that each compound has a different range of water solubility. The range of highly soluble to insoluble compounds, as determined by the water solubility test (calculated in Log S), is insoluble<‐10 poorly soluble<‐ 6, moderately soluble<‐4, soluble<‐2, very soluble<0.^52^, ^53^ Except for ligand 06, which exhibits moderate solubility, the solubility ranges from −3.56 to −3.94 which means most compounds demonstrate good solubility under (Table 3).

**TABLE 3:** Summary of calculation of ADME, and toxicity profile results for selected derivatives.

No	CID	Absorption		Distribution		Metabolism		Excretion		Toxicity	
		Water solubility Log S	Human Intestinal Absorption (%)	VDss (log L/kg)	BBB Permeability	CYP450 1A2 Inhibitor	CYP450 2D6 Substrate	Total Clearance (ml/min/kg)	Renal OCT2 substrate	AMES toxicity	Skin sensitisation
01	6438629	−3.949	93.347	−0.813	0.009	No	No	1.471	No	No	No
02	20849244	−3.949	93.347	−0.813	0.009	No	No	1.471	No	No	No
03	5364256	−3.603	96.141	−0.47	0.21	No	No	1.214	No	No	No
04	10253806	−3.982	66.667	−0.396	−0.931	No	No	1.259	No	No	No
05	71316382	−3.603	96.141	−0.47	0.21	No	No	1.214	No	No	No
06	10427626	−5.991	95.758	0.2	0.227	No	No	1.603	No	No	Yes
07	14443943	−3.565	96.376	−0.457	0.242	No	No	1.214	No	No	No
08	101278260	−3.603	96.141	−0.47	0.21	No	No	1.214	No	No	No

The blood–brain barrier (BBB) and the steady‐state method (VDss) volume of distribution have been chosen as our significant elements for distribution prediction. The VDss values show the degree of drug distribution uniformity between the tissue and blood. When the VDss score is >0.45, it means that the therapeutic molecule is distributed more evenly throughout the body. On the other hand, a lower result—one that is less than −0.15—indicates an uneven distribution of the medication.^54^ The range of the VDss (human) is −0.39 to −0.81. The VDss score of ligand number 06, which is 0.2, showed an excellent distribution pattern; however, the VDss scores of the other derivatives were not satisfactory. By acting as a barrier, the BBB keeps our brain from interacting with outside substances. This suggests that when choosing the best drug‐like compounds, evaluating BBB permeability is a essential factor. The distribution is poor when the BBB permeability score is less than−1. A score of >0.3, on the other hand, denotes suitable BBB permeability.^55^ No ligands showed BBB permeability more significant than 0.3. As well as ligand no 4 showed poor bioavailability, which is −0.931.

The cytochrome P450 (CYP450) family is essential for the conversion of drugs into forms that can be excreted from the body. Inhibition of CYP1A2 may lead to drug interactions due to the irreversible reduction in enzyme activity,^56^ and drugs metabolized by CYP2D6 may increase the incidence of adverse effects due to the polymorphic nature of this cytochrome, resulting in pharmacokinetic differences potentially affecting the therapeutic efficacy and toxicity of the drug.^57^ According to the metabolic profile analysis, no ligand inhibited the CYP450 2D6 substrate, and all ligands demonstrated negative CYP450 1A2 inhibition.

A thorough evaluation of drug excretion is made possible by evaluating total drug clearance, which considers the impact of organic cation transporter 2 (OCT2).^58^ Total clearance includes information on both hepatic and renal clearance, which makes it easier to understand a drug's excretion profile in its entirety.^59^ During our study, all of the selected derivatives showed overall more outstanding clearance scores. Moreover, none of the reported substances were expected to have the potential to be renal OCT2 substrates.

In terms of toxicity, this analysis allows us to identify whether new drugs can trigger allergic reactions such as allergic contact dermatitis, which causes redness, swelling and itching at the affected site and exposes the body to infections that can be fatal for a patient undergoing cancer treatment.^60^

Damage to the genome have significant importance due to its serious and well‐studied effects on human health. Therefore, the AMES test is a bacterial mutation bioassay used to assess the genotoxic potential of a substance, which is crucial for the development of new drugs.^61^, ^62^ Skin sensitisation was also evaluated. In our analysis, no molecule showed toxicity for both properties, except for molecule 6 for skin sensitisation.

### 3.5 MD Simulation Analysis

#### 3.5.1 Root‐mean‐square deviation (RMSD) analysis

**FIGURE 2:**
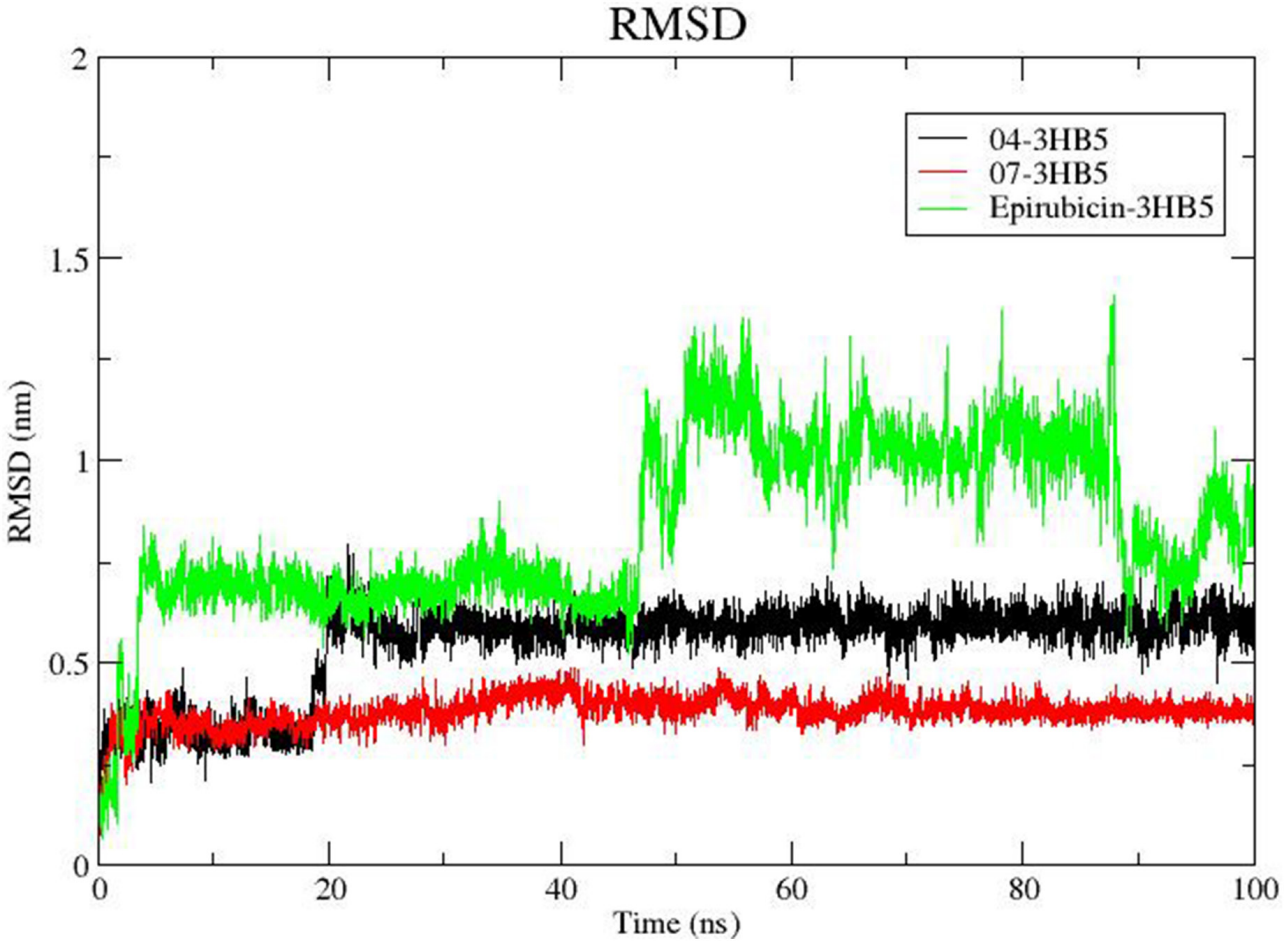
RMSD trajectories of 17beta‐HSD1 (3HB5) complexes with retinoic acid derivatives and Epirubicin over a 100 ns MD simulation. The black curve represents the RMSD for the complex with ligand 04, indicating moderate conformational variability. The red curve corresponds to ligand 07 and shows a remarkably stable complex with minimal RMSD fluctuation. The green curve illustrates the RMSD for the complex of the standard drug Epirubicin, which exhibits greater instability. These patterns reflect the dynamic stability and structural conformation of protein‐ligand interactions over time.

The RMSD analysis demonstrates distinct stability patterns among ligand 04, ligand 07, and the standard Epirubicin in their respective complexes with protein (PDB ID: 3HB5) (depicted in Figure 2). Ligand 07, represented by the red curve, exhibits notable stability with minimal fluctuation around 0.35 nm after an initial rise from 0 nm. In the case of ligand 04 (black curve), the RMSD remains steady around 0.35 nm until 20 ns, followed by a subsequent increase to approximately 0.65 nm, maintaining this level until the end of the 100 ns simulation. Meanwhile, the RMSD of the standard Epirubicin (depicted in green) initially rises during the first 5 ns, stabilizes around 0.7 nm until 43 ns, experiences another increase to 1.1 nm until around 84 ns, and eventually drops to about 0.75 nm with minimal fluctuation until 100 ns. Significantly, the introduction of ligand 07 does not compromise the stability of the protein structure, as evidenced by consistent conformations throughout the 100 ns simulation, however, high instability was observed in the case of 03‐3HB5 and Epirubicin‐3HB5. This means that ligand 07 is more stable than ligand 04 and the standard Epirubicin in the protein target.

#### 3.5.2 Root‐mean‐square fluctuation (RMSF)

**FIGURE 3:**
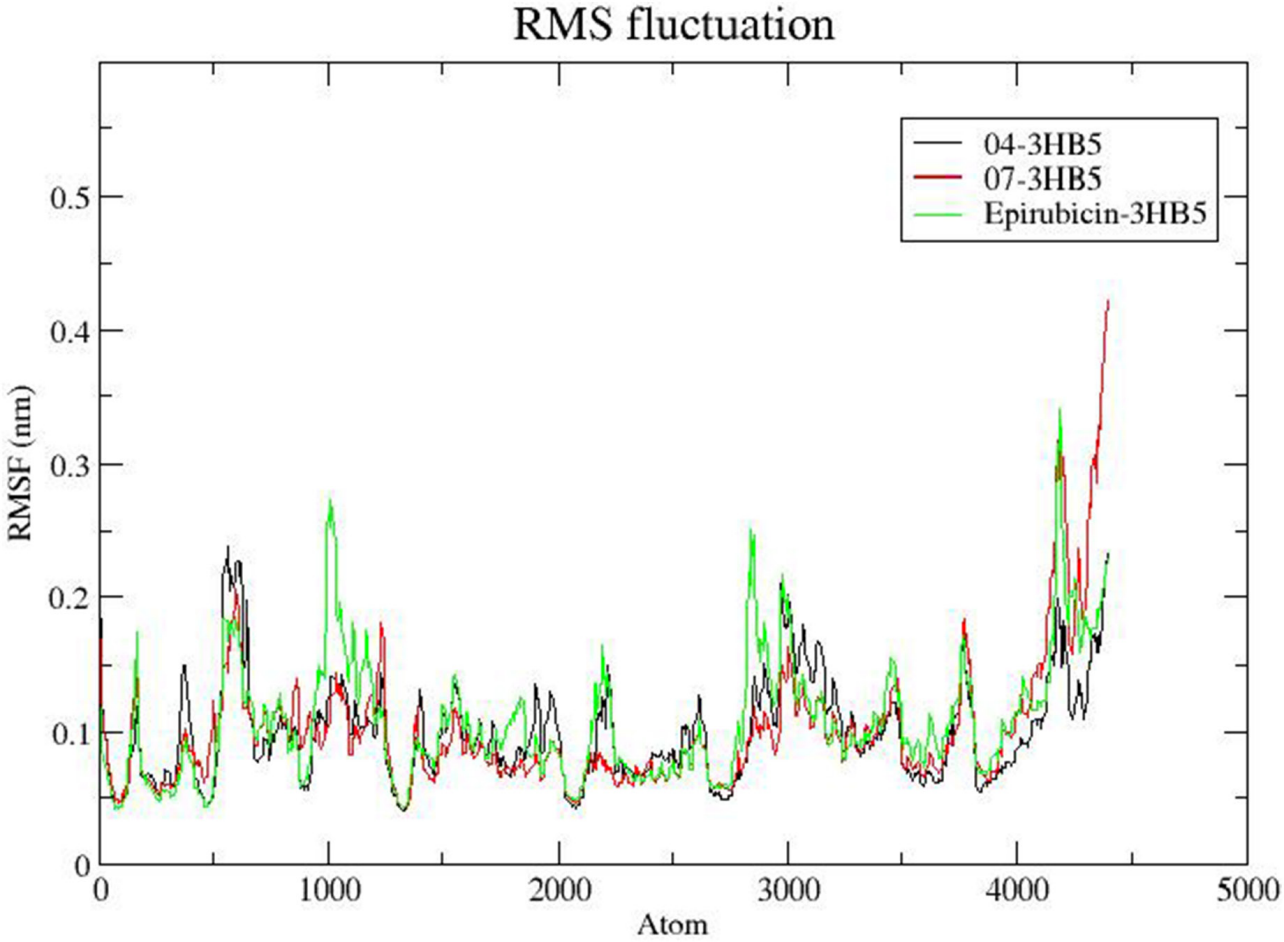
Protein Cα‐RMSFs during 100 ns MD simulation with (A) Ligand 04 (B) Ligand 07 (C) Standard Epirubicin.

The protein backbone's RMSF data were utilized to generate visual representations of the average fluctuation for all amino acid residues in each complex, as illustrated in Figure 3 for a 100 ns MD trajectory. Upon analysing the RMSF of the alpha carbon (Cα) in the 04‐3HB5 complex, it was observed that nearly all atom positions experienced deviations of less than 2.0 Å, In the case of 07‐3HB5 complex, the highest values of RMSF (>2.0 Å) were observed. Likewise, in the standard Epirubicin‐3HB5 complex, more amino acid residue atoms were observed to have RMSF values higher than 2.0 Å. Notably, these residue atoms with higher RMSF values (>2.0 Å) are located outside the protein binding sites, suggesting that they might not significantly impact the ligand binding process. These flexible residues within the loop region of the protein tend to fluctuate more than other residues. These residues at the protein's terminus demonstrate heightened flexibility, reactivity, and freedom of movement compared to other amino acid residues due to their positioning at the end of the protein sequence.

#### 3.5.3 Hydrogen bond analysis

**FIGURE 4:**
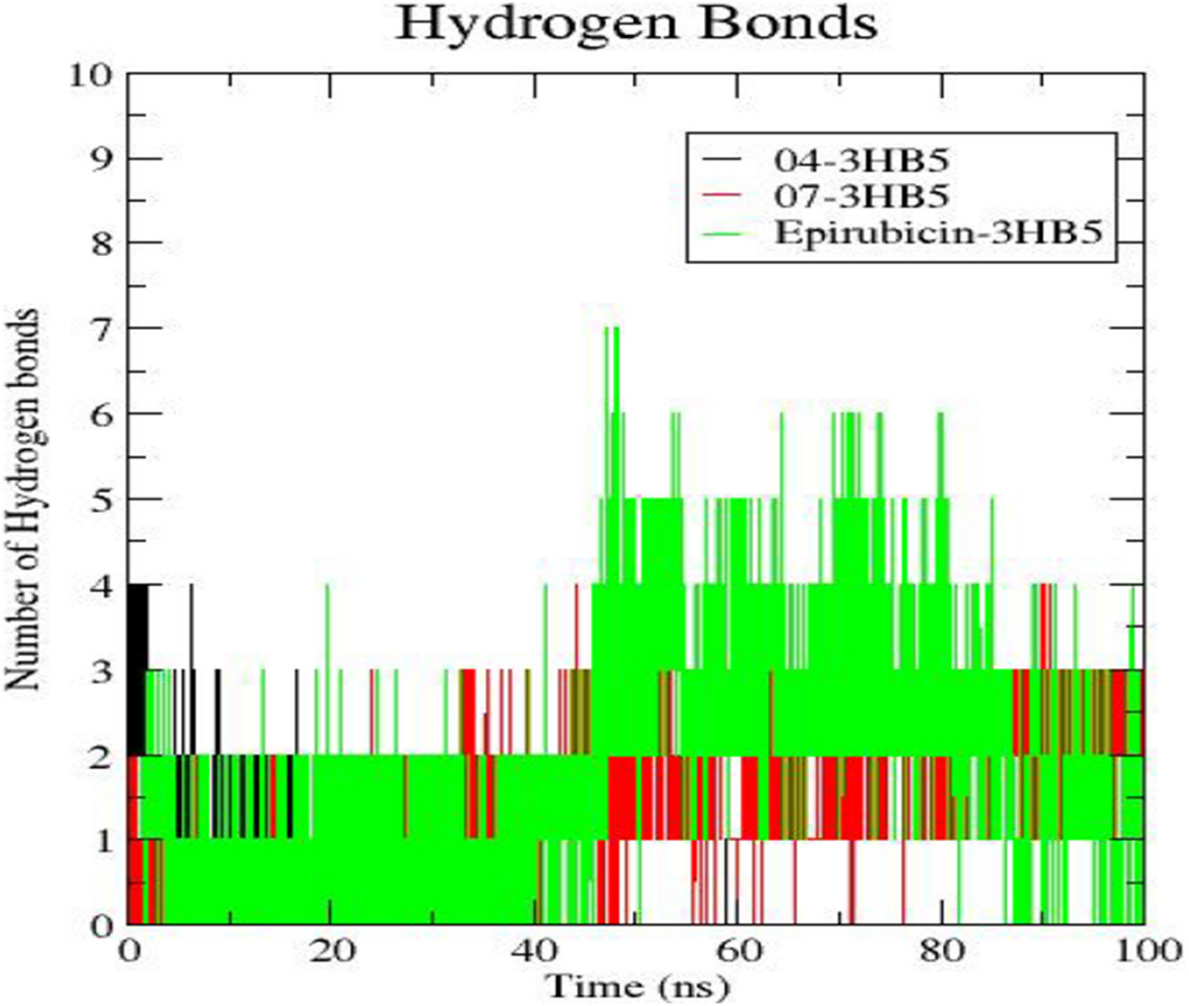
Hydrogen bond formation within 100 ns (A) Ligand 04‐3HB5 complex (B) Ligand 07‐3HB5 complex (C) Epirubicin‐3HB5 complex.

The robustness of the highest‐docked complexes (ligand 04 and 07) is supported by the number of intermolecular hydrogen bonds formed throughout the 100 ns simulation. Figure 4 provides a detailed account of the hydrogen bonds in both the highest‐docked complex (ligand 04 and 07) and the standard Epirubicin complex. The plot illustrating the number of hydrogen bonds over time (100 ns) for the entire simulation demonstrates that initially, up to around 20 ns, the 04‐3HB5 complex exhibits 1 to 4 bonds. However, from 20 ns onwards, the complex shows weak intermolecular interactions. In the case of 07‐3HB5, the number of hydrogen bonds is more consistent initially, up to around 32 ns, at 1 and 2, while from 32 ns onward, the number becomes more consistently 3. On the contrary, the Epirubicin complex shows the highest number of hydrogen bonds (7) at around 46 ns. However, these interactions lack regularity and appear disrupted, with two to seven hydrogen bonds observed intermittently. Only two to five bonds remain stable at most time points.

#### 3.5.4 Dynamic Cross‐Correlation Matrix (DCCM) Analysis

**FIGURE 5:**
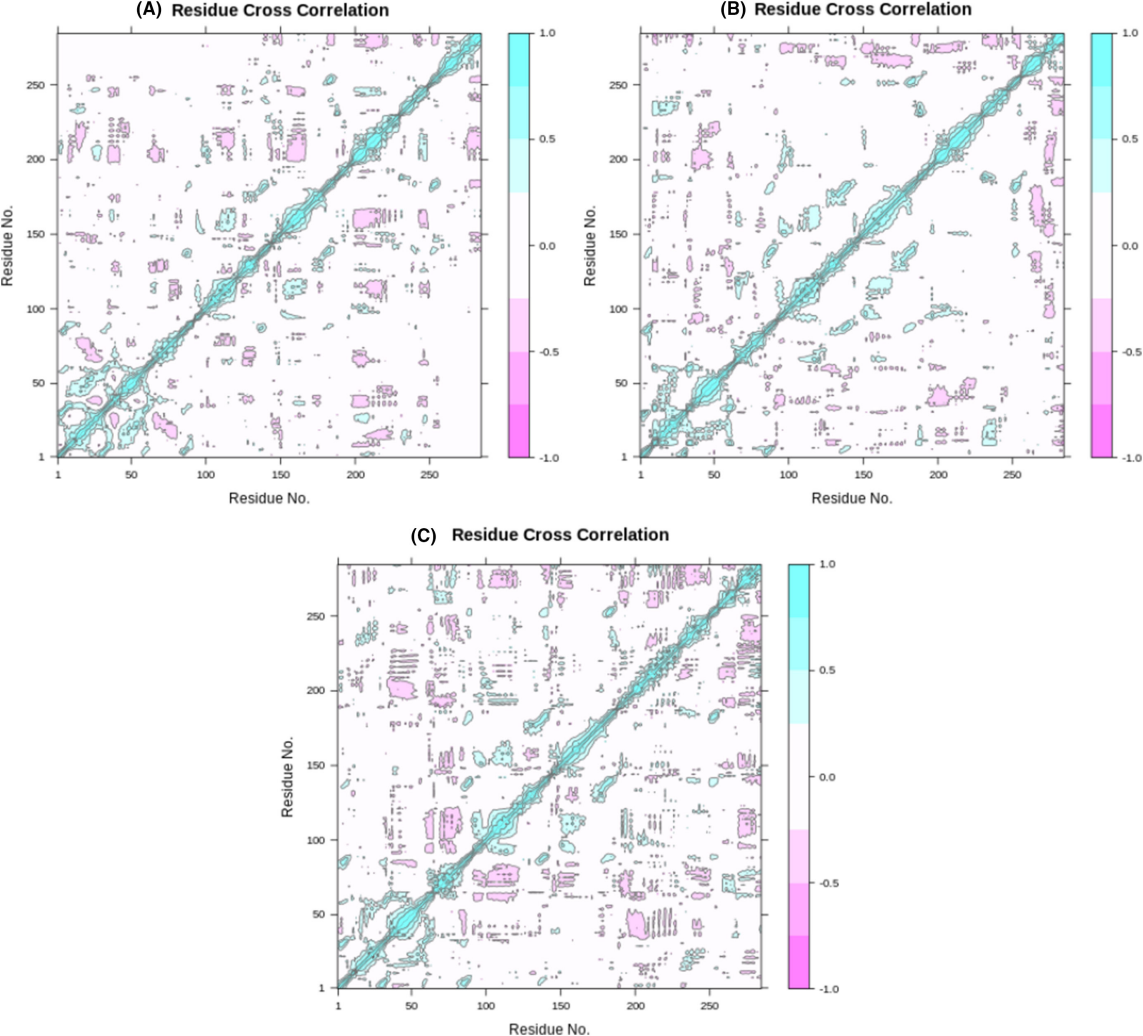
Cα‐Residues Cross‐Correlation Profiles for the Protein‐Ligand Complexes: (A) Ligand 04‐3HB5 complex (B) Ligand 07‐3HB5 complex (C) Epirubicin‐3HB5 complex. The correlation coefficients, ranging from −1 to 1, provided insights into whether residues exhibited positive or negative relationships in their motions. A positive correlation suggested that residues moved in the same direction, whereas a negative correlation indicated movements in opposite directions.

The DCCM analysis was conducted on all carbon atoms within the 04‐3HB5 complex, 07‐3HB5 complex, and standard Epirubicin‐3HB5 complex using 100 ns simulated trajectories (Figure 5A–C). The DCCM presented a correlation scale ranging from −1.0 to 1.0, where the former was depicted by a dark purple shade and the latter by a dark blue shade. Distinct colour shades denoted varying degrees of correlation between residues, with deeper colours indicating stronger associations. The correlation coefficients, spanning from −1 to 1, shed light on whether residues exhibited positive or negative relationships in their motions. A positive correlation suggested that residues moved in the same direction, while a negative correlation indicated opposite movements. Upon analysing the DCCM diagrams of the three systems, similar correlations were observed, suggesting that the atomic motions in these systems closely resemble each other.^63^, ^64^

#### 3.5.5 Principal components Analysis (PCA)

**FIGURE 6:**
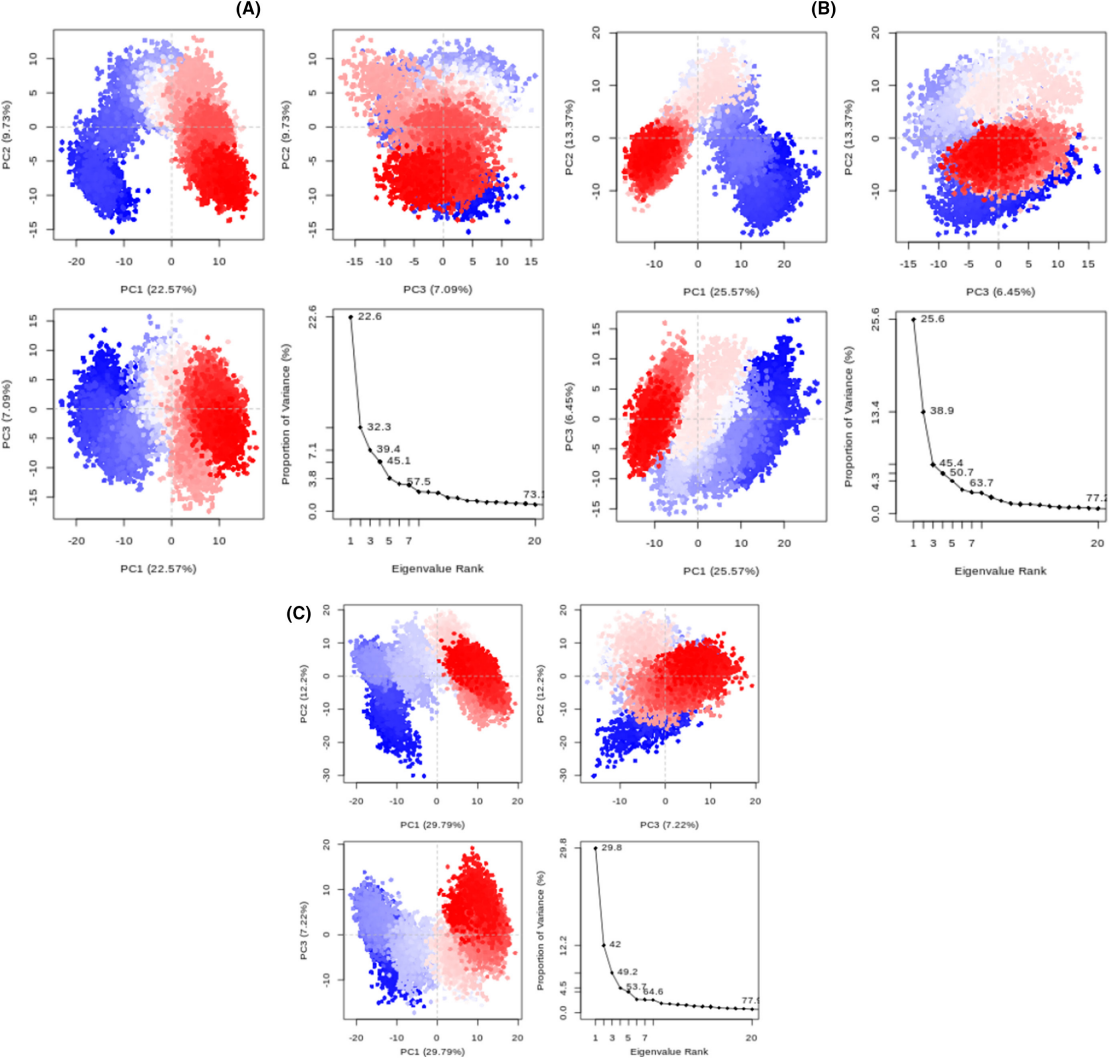
PCA plots of the protein backbone in the complex, comprising graphs of PC2 vs PC1, and an eigenvalue rank plot with the cumulative variance annotated for each data point. (A) Ligand 04‐3HB5 complex (B) Ligand 07‐3HB5 complex (C) Epirubicin‐3HB5 complex. The colour gradient transitioned from blue to white to red, signifying the progression of the simulation, with blue representing the initial timestep, white indicating the intermediate timestep, and red signifying the final timestep.

**FIGURE 7:**
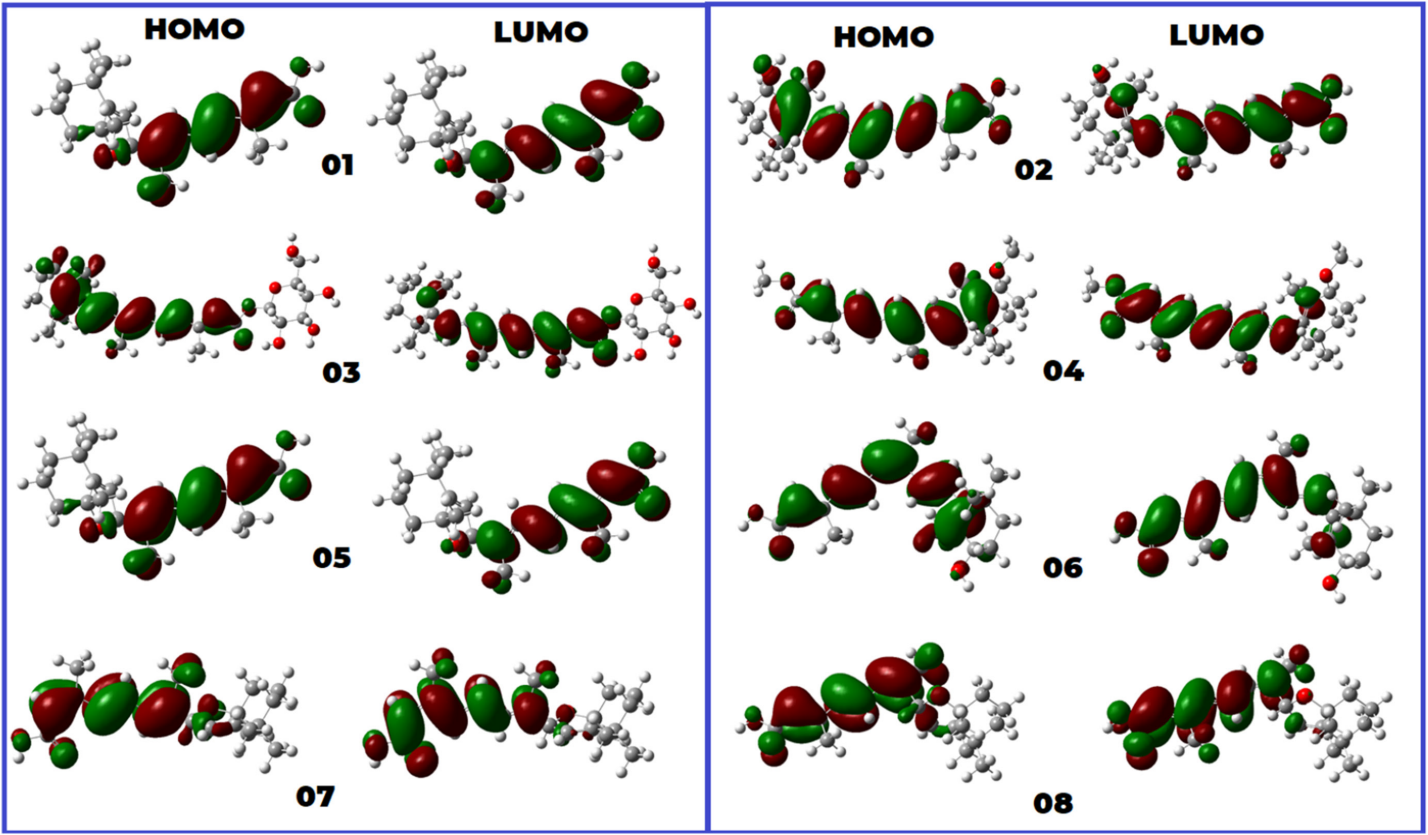
HOMO‐LUMO diagram.

**FIGURE 8:**
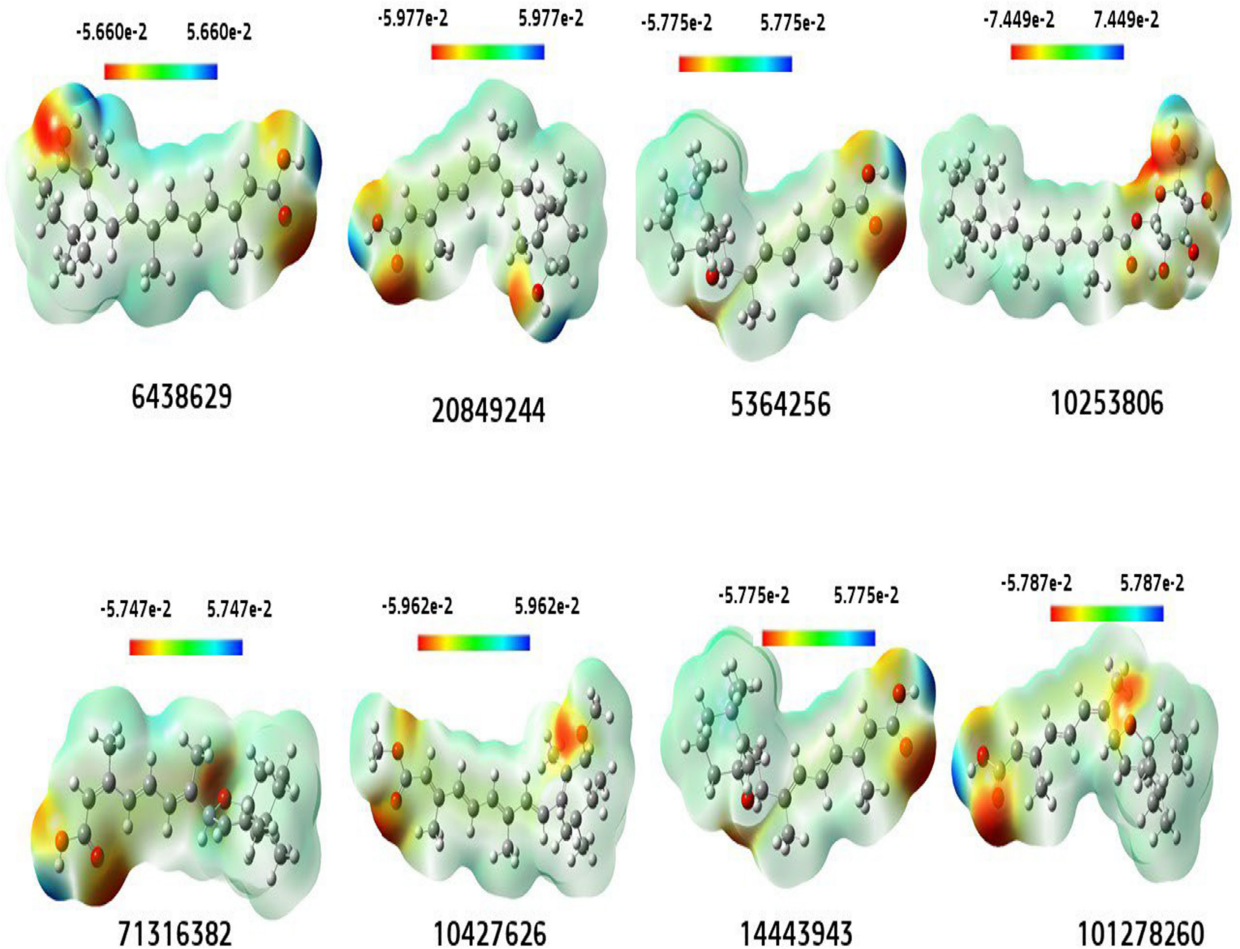
MEP illustration, and charge distribution mapping.

As illustrated in Figure 8, the initial 20 principal components (PCs) of the C‐alpha backbone of the protein in the 04‐3HB5 complex, 07‐3HB5 complex, and Epirubicin‐3HB5 complex systems collectively explained 73%, 77%, and 77% of the total variance, respectively. This implies that, when compared to the standard ‐3HB5 complex, the 07‐3HB5 complex system demonstrates a similar phase area and flexibility while the 04‐3HB5 complex system displayed a smaller phase area and lower flexibility in performance. In the 04‐3HB5 complex, PC1 and PC2 contributed 22.57% and 9.73% to the variance, respectively, while in the 07‐3HB5 complex system, PC1 and PC2 contributed 25.57% and 13.37%, respectively. In the standard Epirubicin‐3HB5 complex, PC1 and PC2 contributed 29.79% and 12.20% to the total variance, respectively. This indicates that the first two eigenvectors (PC1 and PC2) play a highly indicative role in the conformational state transformation. To depict the conformational states of the two systems in a specific subspace, we projected the data onto the PC1 and PC2 axes, creating PCA scatter plots (Figure 7). In the scatter diagram, the red dot signifies the stable conformational state, the blue dot represents the unstable conformational state, and the intermediate state between the two conformations is denoted by white dots (Figure 6).

### 3.6 Frontier molecular orbital and molecular properties analysis

The HOMO‐LUMO energies, hardness, softness, and energy gap of all compounds are shown in Table 4. The statistical profiles have been calculated using the DFT B3LYP/6‐31G function. The energy difference between the HOMO and the LUMO is a commonly employed metric for evaluating the chemical reactivity of molecules. If the energy gap between the HOMO and LUMO gap of a molecule is large, it indicates that the molecule is chemically more stable and less reactive.^65^, ^66^ The principal cause of this phenomenon is the obstruction of the electronic transition, which is attributed to a substantial energy disparity between the ground and excited states. Typically, a molecule exhibiting a small HOMO–LUMO gap is indicative of a high degree of stability. The values of chemical hardness, softness, and chemical potential depend on the energy levels of the highest occupied molecular orbital (HOMO) and the lowest unoccupied molecular orbital (LUMO). The data in Table 4 demonstrates that the HOMO–LUMO gaps of the compounds under investigation fall within the range of 2.9154–3.774 eV. Among the compounds reported, 03 and 02 had the smallest HOMO–LUMO energy gap, measuring between 2.9154 and 3.105 eV. Conversely, ligand 01 exhibited the highest level of hardness and the most extensive energy gap. Compounds with higher hardness values show more resistance to changes in electron configuration at the molecular level. The obtained hardness data indicate that it may have taken a long time for the specimens to break down after they entered the physiological system. Furthermore, it was discovered that the ligand 03 exhibits molecules with a maximum softness value of 0.686, suggesting a greater propensity for dissolving of this medication. The HOMO–LUMO data and Frontier molecular orbital diagram can be found in Figures 7 and 4, respectively.

**TABLE 4:** Chemical reactivity and molecular properties data.

No	PubChem CID	HOMO	LUMO	GAP	Hardness	Softness
01	5364256	−5.821334802	−2.0465693	3.774765502	1.887382751	0.5298342
02	6438629	−5.4697635	−2.3641264	3.105637068	1.552818534	0.6439902
03	10253806	−5.47166831	−2.556238	2.915430312	1.457715156	0.6860050
04	10427626	−5.306495114	−2.170653378	3.135841736	1.567920868	0.6377872
05	14443943	−5.821334802	−2.046569394	3.774765408	1.887382704	0.5298342
06	20849244	−5.375884184	−2.21636853	3.159515740	1.579757870	0.6330083
07	71316382	−5.737795804	−1.99731676	3.740479044	1.870239522	0.5346908
08	101278260	−5.78922535	−2.043304026	3.745921324	1.872960662	0.5339140

### 3.7 Molecular of electrostatic potential (MEP) charge distribution mapping

The MEP includes a compatibility feature that identifies the most favourable regions for the targeted electrophile and nucleophile of charged area molecules on organic materials. The MEP elucidates the biological mechanism and the coupling or interaction of H‐bonding, which is essential in the study of characterising organic compounds. The electrostatic potential map provides a method for assessing the interactions among various molecule geometries.^67^ The present investigation involved the simulation of the electrostatic potential map of the specific chemical using the B3LYP method, employing a 6‐31G basis set. The structure was then optimized, yielding the results shown in Figure 8. It visually represents the molecule structure and size and the regions of positive, negative, and neutral electrostatic probability through colour differentiation. In addition, it is also an essential method for identifying the correlation between physicochemical properties and the structure of the specific drug. The potential of the attacking area decreases in the following sequence: blue, red, and white. The white section represents the neutral area where no attrack takes place. The red colour indicate regions of high electron saturation, suggesting that electrophiles are likely to rapidly attack these areas. The lowest electron density surface is indicated by the blue colour, making it susceptible to nucleophilic attack. In all the compounds studied, it was observed that the nucleophilic sites were mainly concentrated on the electron‐negative oxygen atoms, and mostly on the oxygen atoms located in the aromatic ring. Meanwhile, the electrophilic attack sites were localized on the hydrogen of the hydroxyl group (H_2_).

## 4 DISCUSSION

In our study, a series of computational methods were used to evaluate the efficacy of retinoic acid derivatives as inhibitors of 17beta‐hydroxysteroid dehydrogenase type 1. Among the analysed ligands, some candidates proved to be particularly promising as they exhibited excellent binding affinities, favourable pharmacokinetic profiles, and remarkable stability in MD simulations.

Initially, it should be clarified that the use of Epirubicin hydrochloride as a reference compound in this study serves as a strategic standard to **assess** the therapeutic potential of novel retinoic acid derivatives compared to an established chemotherapeutic agent in the treatment of BC, despite the different mechanism of action of 17beta‐HSD1 inhibitors. The inclusion of Epirubicin facilitates a comprehensive evaluation of the new agents and enables a comparison that goes beyond specific enzyme inhibition to include a more comprehensive assessment of anticancer efficacy, pharmacokinetic profiles, and safety. This comparison aims to highlight the potential multifaceted benefits of the newly identified inhibitors, positioning them in the current therapeutic landscape and highlighting their potential advantages over conventional treatments. Considering the complex nature of cancer treatment and the necessity for a comprehensive approach to drug assessment, using epirubicin as a benchmark offers a wider view on the comparative effectiveness and safety of novel agents.

The PASS prediction indicated that all molecules are highly reactive against neoplastic diseases. The molecular docking study confirms this finding. The molecules are particularly promising, with compounds 03, 04 and 07 having the highest binding affinity and even exceeding the affinity of standard Epirubicin, indicating a significant potential to disrupt the enzymatic pathways that contribute to the development of BC.

The ADMET profile of the molecules is adequate, but there are some limitations. The molecule 06 has a low solubility under physiological conditions compared to the other molecules and is associated with skin sensitisation. This applies to drugs that are applied directly to the skin, indicating a possible allergic reaction that could pose indirect risks to the life of the treated patient.

Blood–brain barrier permeability issues are addressed in our study and point to the need for new drug delivery strategies for BC brain metastases (BCBM), as none of the ligands showed the ability to cross the barrier. The results indicate promising pharmacokinetic profiles, however, their efficacy would be enhanced by overcoming the BBB and the resulting restrictive blood‐tumour barrier (BTB) to deliver therapeutic agents into brain tumours. The use of advanced targeted delivery systems, such as nanoparticles, may be a viable solution to the poor levels of BBB vascular permeability. Approaches aimed at improving this property could significantly contribute to improving treatment outcomes in BCBM by circumventing the barriers that limit the bioavailability of current drugs.

In addition, the molecule 04 has a low intestinal absorption, which is below the ideal value, which would mean that a higher dose of the drug is required to achieve its therapeutic effect.^68^ However, MD simulation of this ligand confirmed a stable interaction with the target protein, supporting its suitability for in vivo and in vitro validation studies. Analysis of hydrogen bonds and hydrophobic interactions highlights the potential of these interactions to contribute to the ligand's efficacy as a 17beta‐HSD1 inhibitor.

However, the path from computational models to clinically approved drugs face an obstacle. One of the main limitations of the study arises in the simplifications inherent in computational models. These cannot fully capture the complex dynamics of biological systems, so experimental validation is required to confirm the therapeutic potential of these ligands. While the pharmacokinetic profiles are promising, they should be investigated through in vitro and in vivo studies to assess the behaviour of the compounds in a living organism, including their metabolism, bioavailability, and potential toxicity.

This study serves as a solid foundation for the search for new treatment options and highlights the significant impact of computational drug design on the identification of promising therapeutic candidates, as already observed in several studies in the literature,^69^, ^70^, ^71^ also in the fight against COVID‐19 in 2020.^72^

## 5 CONCLUSION

In conclusion, our study introduces a new group of retinoic acid derivatives identified by computer‐aided drug design as possible 17beta‐HSD1 inhibitors and provides a direction for further experimental research into how to the treat of BC. In exclusive, ligands 4 and 7 showed a higher binding affinity than the reference compound (Epirubicin hydrochloride), indicating a potential advantage in therapeutic efficacy. These computational results provide a starting point for future experimental validation to confirm their potential as viable therapeutic agents. Our study shows the potential of in silico study model in the initial stages of drug discovery, highlighting the demand for continued research in this field to overcome the obstacles associated with treating BC.

## AUTHOR CONTRIBUTIONS

**Md. Rezaul Islam:** Conceptualization (lead); data curation (equal); formal analysis (equal); methodology (equal); visualization (equal); project administration (equal); writing – original draft (lead). **Jehad Zuhair Tayyeb:** Writing – original draft (equal); Validation; investigation (equal); methodology (lead); visualization (equal); **Hridoy Kumar Paul:** Conceptualization (equal); data curation (equal); formal analysis (equal); methodology (equal); writing – original draft (equal); visualization (equal). **Mirza Nafeul Islam:** Conceptualization (equal); methodology (equal); formal analysis (equal); project administration (equal); validation (equal); visualization (equal); writing – original draft (equal). **Gbolahan Oladipupo Oduselu:** Data curation (equal); investigation (equal); project administration (equal); resources (equal); software (equal); validation (equal); methodology (supporting). **Imren Bayıl:** Data curation (equal); formal analysis (equal); methodology (equal); software(equal); resources (equal); visualization (equal); writing – original draft (supporting). **Magda H. Abdellattif:** software (equal); validation (equal); visualization (equal); investigation (equal); writing – review and editing (equal); supervision (equal). **Khairia Mohammed Al‐Ahmary:** Formal analysis (equal); validation (equal); writing – original draft (supporting). **Saedah R. Al‐Mhyawi:** Data curation (equal); formal analysis (equal); validation (equal); investigation (equal); project administration (equal); visualization (equal). **Magdi E. A. Zaki:** Conceptualization (equal); Writing – review and editing (equal); supervision (Lead); validation (equal).

## FUNDING INFORMATION

This research did not receive any specific funding and has no relationship with any commercial entity or individual that may create a potential conflict of interest.

## CONFLICT OF INTEREST STATEMENT

The authors declare no conflict of interest exists.

## Supporting information


Table S1:

## Data Availability

Data are available in the manuscript.
