# Supplementary material for: In silico analysis of potential inhibitors for breast cancer targeting 17beta‐hydroxysteroid dehydrogenase type 1 (17beta‐HSD1) catalyses
Source: J Cell Mol Med. 2024 Aug 12;28(15):e18584. doi: 10.1111/jcmm.18584 (PMC11319393; doi:10.1111/jcmm.18584)
Supplement: Supplementary file 1 — Table S1: [file JCMM-28-e18584-s001.docx]

**Supplementary Table S1:** Biological pass prediction spectrum computation of Retinoic Acid derivatives.

| **No.** | **CID** | **Antineoplastic** | | **Antibacterial** | | **Antiviral (Influenza)** | | |
| --- | --- | --- | --- | --- | --- | --- | --- | --- |
|  |  | **Pa** | **Pi** | **Pa** | **Pi** | **Pa** | | **Pi** |
| 1 | 444795 | 0.874 | 0.005 | 0.334 | 0.048 | 0.262 | | 0.120 |
| 2 | 449171 | 0.874 | 0.005 | 0.334 | 0.048 | 0.262 | | 0.120 |
| 3 | 5282379 | 0.874 | 0.005 | 0.334 | 0.048 | 0.262 | | 0.120 |
| 4 | 60164 | 0.297 | 0.154 | 0.163 | 0.152 | 0.635 | | 0.010 |
| 5 | 82146 | 0.581 | 0.049 |  |  | 0.226 | | 0.160 |
| 6 | 5284513 | 0.754 | 0.018 | 0.293 | 0.063 | 0.204 | | 0.191 |
| 7 | 5282375 | 0.729 | 0.021 | 0.217 | 0.103 | 0.204 | | 0.192 |
| 8 | 638034 | 0.880 | 0.005 | 0.348 | 0.044 |  | |  |
| 9 | 11518241 |  |  | 0.173 | 0.143 | 0.220 | | 0.168 |
| 10 | 108143 | 0.274 | 0.169 | 0.226 | 0.097 | 0.428 | | 0.038 |
| 11 | 5288209 | 0.859 | 0.006 | 0.303 | 0.059 | 0.210 | | 0.182 |
| 12 | 6438629 | 0.911 | 0.005 | 0.395 | 0.031 | 0.323 | | 0.076 |
| 13 | 6437063 | 0.845 | 0.007 | 0.346 | 0.044 | 0.277 | | 0.106 |
| 14 | 447276 | 0.844 | 0.007 | 0.351 | 0.043 | 0.251 | | 0.131 |
| 15 | 5363137 | 0.801 | 0.012 | 0.234 | 0.093 | 0.209 | | 0.183 |
| 16 | 9904203 | 0.657 | 0.034 | 0.311 | 0.056 | 0.219 | | 0.170 |
| 17 | 6437836 | 0.809 | 0.010 | 0.469 | 0.019 | 0.283 | | 0.101 |
| 18 | 6314185 | 0.741 | 0.020 | 0.239 | 0.089 |  | |  |
| 19 | 6419708 | 0.874 | 0.005 | 0.334 | 0.048 | 0.262 | | 0.120 |
| 20 | 5281877 | 0.896 | 0.005 | 0.605 | 0.009 | 0.615 | | 0.012 |
| 21 | 5378821 | 0.881 | 0.005 | 0.284 | 0.066 | 0.207 | | 0.187 |
| 22 | 9863341 | 0.488 | 0.075 | 0.161 | 0.154 | 0.236 | | 0.148 |
| 23 | 9864378 | 0.544 | 0.059 | 0.202 | 0.116 | 0.688 | | 0.006 |
| 24 | 9796370 | 0.874 | 0.005 | 0.334 | 0.048 | 0.262 | | 0.120 |
| 25 | 6506224 | 0.874 | 0.005 | 0.300 | 0.060 | 0.306 | | 0.085 |
| 26 | 20849244 | 0.911 | 0.005 | 0.395 | 0.031 | 0.323 | | 0.076 |
| 27 | 5288173 | 0.857 | 0.006 | 0.281 | 0.067 |  | |  |
| 28 | 6435504 | 0.835 | 0.008 | 0.387 | 0.033 | 0.291 | | 0.095 |
| 29 | 6436116 | 0.730 | 0.021 |  |  | 0.244 | | 0.139 |
| 31 | 10335106 | 0.833 | 0.008 | 0.392 | 0.032 | 0.207 | | 0.187 |
| 32 | 6505275 | 0.825 | 0.009 | 0.267 | 0.074 | 0.252 | | 0.129 |
| 33 | 10266931 | 0.801 | 0.012 | 0.351 | 0.043 | 0.217 | 0.172 | |
| 34 | 6505370 | 0.853 | 0.007 | 0.258 | 0.079 | 0.261 | | 0.120 |
| 34 | 6436049 | -- | -- | 0.172 | 0.144 | 0.279 | | 0.104 |
| 35 | 11130378 | 0.803 | 0.011 | 0.486 | 0.018 | 0.249 | | 0.133 |
| 36 | 44579059 | 0.612 | 0.043 | 0.378 | 0.036 | 0.368 | | 0.057 |
| 37 | 10448377 | 0.881 | 0.005 | 0.284 | 0.066 | 0.207 | | 0.187 |
| 38 | 5364256 | 0.930 | 0.005 | 0.430 | 0.024 | 0.218 | | 0.171 |
| 39 | 11174913 | 0.902 | 0.005 | 0.374 | 0.037 | 0.271 | | 0.111 |
| 40 | 10381862 | 0.854 | 0.006 | 0.299 | 0.060 | 0.220 | | 0.168 |
| 41 | 11130378 | 0.803 | 0.011 | 0.486 | 0.018 | 0.249 | | 0.133 |
| 42 | 44579059 | 0.612 | 0.043 | 0.378 | 0.036 | 0.368 | | 0.057 |
| 43 | 10448377 | 0.881 | 0.005 | 0.284 | 0.066 | 0.207 | | 0.187 |
| 44 | 11174913 | 0.902 | 0.005 | 0.374 | 0.037 | 0.271 | | 0.111 |
| 45 | 10381862 | 0.854 | 0.006 | 0.299 | 0.060 | 0.220 | | 0.168 |
| 46 | 25054592 | 0.836 | 0.008 | 0.193 | 0.124 |  | |  |
| 47 | 303533 | 0.860 | 0.006 |  |  | 0.207 | | 0.186 |
| 48 | 6438744 | 0.692 | 0.027 | 0.176 | 0.140 |  | |  |
| 49 | 15125876 | 0.868 | 0.005 | 0.229 | 0.095 |  | |  |
| 50 | 12358676 | 0.874 | 0.005 | 0.334 | 0.048 | 0.262 | | 0.120 |
| 51 | 9995780 | 0.882 | 0.005 | 0.390 | 0.032 | 0.337 | | 0.069 |
| 52 | 9972939 | 0.845 | 0.007 | 0.346 | 0.044 | 0.277 | | 0.106 |
| 53 | 10881132 | 0.874 | 0.005 | 0.334 | 0.048 | 0.262 | | 0.120 |
| 54 | 10253806 | 0.912 | 0.005 | 0.581 | 0.010 | 0.557 | | 0.016 |
| 55 | 10881705 | 0.751 | 0.018 | 0.190 | 0.127 |  | |  |
| 56 | 6437087 | 0.845 | 0.007 | 0.346 | 0.044 | 0.277 | | 0.106 |
| 57 | 102461799 | 0.911 | 0.005 | 0.395 | 0.031 | 0.323 | | 0.076 |
| 58 | 6438977 | 0.825 | 0.009 | 0.267 | 0.074 | 0.252 | | 0.129 |
| 59 | 9883252 | 0.887 | 0.005 | 0.306 | 0.058 | 0.224 | | 0.163 |
| 60 | 627275 | 0.896 | 0.005 | 0.605 | 0.009 | 0.615 | | 0.012 |
| 61 | 71316382 | 0.930 | 0.005 | 0.430 | 0.024 | 0.218 | | 0.171 |
| 62 | 71316381 | 0.801 | 0.012 | 0.234 | 0.093 | 0.209 | | 0.183 |
| 63 | 21724639 | 0.881 | 0.005 | 0.284 | 0.066 | 0.207 | | 0.187 |
| 64 | 9822324 | 0.856 | 0.006 | 0.407 | 0.028 |  | |  |
| 65 | 46782872 | 0.874 | 0.005 | 0.334 | 0.048 | 0.262 | | 0.120 |
| 66 | 122173819 | 0.852 | 0.007 | 0.373 | 0.037 | 0.386 | | 0.051 |
| 67 | 72941664 | 0.791 | 0.013 | 0.417 | 0.026 | 0.257 | | 0.124 |
| 68 | 20839151 | 0.889 | 0.005 | 0.317 | 0.054 | 0.244 | | 0.138 |
| 69 | 16061318 | 0.836 | 0.008 | 0.287 | 0.065 | 0.205 | | 0.189 |
| 70 | 92854578 | 0.801 | 0.012 | 0.234 | 0.093 | 0.209 | | 0.183 |
| 71 | 16061319 | 0.836 | 0.008 | 0.287 | 0.065 | 0.205 | | 0.189 |
| 72 | 16061321 | 0.824 | 0.009 | 0.347 | 0.044 | 0.227 | | 0.159 |
| 73 | 6913131 | 0.874 | 0.005 | 0.334 | 0.048 | 0.262 | | 0.120 |
| 74 | 46782171 | 0.910 | 0.005 | 0.373 | 0.037 | 0.307 | | 0.084 |
| 75 | 6444994 | 0.844 | 0.007 | 0.336 | 0.047 | 0.205 | | 0.189 |
| 76 | 11724060 | 0.853 | 0.007 | 0.258 | 0.079 | 0.261 | | 0.120 |
| 77 | 6914379 | 0.874 | 0.005 | 0.334 | 0.048 | 0.262 | | 0.120 |
| 78 | 6506621 | 0.879 | 0.005 | 0.430 | 0.024 | 0.393 | | 0.049 |
| 79 | 16061320 | 0.824 | 0.009 | 0.347 | 0.044 | 0.227 | | 0.159 |
| 80 | 104857 | 0.845 | 0.007 | 0.346 | 0.044 | 0.277 | | 0.106 |
| 81 | 9839397 | 0.874 | 0.005 | 0.334 | 0.048 | 0.262 | | 0.120 |
| 82 | 10740535 | 0.870 | 0.005 | 0.465 | 0.020 |  | |  |
| 83 | 162930432 | 0.899 | 0.005 | 0.551 | 0.012 | 0.500 | | 0.023 |
| 84 | 10427626 | 0.923 | 0.005 | 0.329 | 0.050 | 0.248 | | 0.134 |
| 85 | 10457706 | 0.813 | 0.010 | 0.308 | 0.057 | 0.266 | | 0.116 |
| 86 | 15384868 | 0.863 | 0.006 | 0.309 | 0.057 | 0.235 | | 0.149 |
| 87 | 71752029 | 0.881 | 0.005 | 0.284 | 0.066 | 0.207 | | 0.187 |
| 88 | 94005047 | 0.801 | 0.012 | 0.234 | 0.093 | 0.209 | | 0.183 |
| 89 | 10471728 | 0.854 | 0.006 | 0.299 | 0.060 | 0.220 | | 0.168 |
| 90 | 6439661 | 0.874 | 0.005 | 0.334 | 0.048 | 0.262 | | 0.120 |
| 91 | 86583356 | 0.890 | 0.005 | 0.627 | 0.007 | 0.644 | | 0.010 |
| 92 | 6913160 | 0.874 | 0.005 | 0.334 | 0.048 | 0.262 | | 0.120 |
| 93 | 56940677 | 0.896 | 0.005 | 0.605 | 0.009 | 0.615 | | 0.012 |
| 94 | 14443943 | 0.930 | 0.005 | 0.430 | 0.024 | 0.218 | | 0.171 |
| 95 | 54305565 | 0.874 | 0.005 | 0.334 | 0.048 | 0.262 | | 0.120 |
| 96 | 6441098 | 0.874 | 0.005 | 0.300 | 0.060 | 0.306 | | 0.085 |
| 97 | 6913136 | 0.874 | 0.005 | 0.334 | 0.048 | 0.262 | | 0.120 |
| 98 | 10450945 | 0.818 | 0.010 | 0.249 | 0.084 | 0.221 | | 0.168 |
| 99 | 45039634 | 0.845 | 0.007 | 0.346 | 0.044 | 0.277 | | 0.106 |
| 100 | 131953110 | 0.825 | 0.009 | 0.384 | 0.034 | 0.409 | | 0.044 |
| 101 | 54501507 | 0.834 | 0.008 | 0.269 | 0.073 | 0.236 | | 0.148 |
| 102 | 11211496 | 0.855 | 0.006 | 0.341 | 0.046 | 0.277 | | 0.106 |
| 103 | 131953106 | 0.892 | 0.005 | 0.423 | 0.025 | 0.466 | | 0.029 |
| 104 | 131953108 | 0.882 | 0.005 | 0.392 | 0.032 | 0.387 | | 0.051 |
| 105 | 57369884 | 0.910 | 0.005 | 0.373 | 0.037 | 0.307 | | 0.084 |
| 106 | 101849851 | 0.864 | 0.006 | 0.359 | 0.041 | 0.338 | | 0.069 |
| 107 | 11724047 | 0.854 | 0.006 | 0.299 | 0.060 | 0.220 | | 0.168 |
| 108 | 11225333 | 0.841 | 0.008 | 0.349 | 0.044 | 0.253 | | 0.129 |
| 109 | 71751404 | 0.845 | 0.007 | 0.346 | 0.044 | 0.277 | | 0.106 |
| 110 | 46782029 | 0.845 | 0.007 | 0.346 | 0.044 | 0.277 | | 0.106 |
| 111 | 101278260 | 0.930 | 0.005 | 0.430 | 0.024 | 0.218 | | 0.171 |
| 112 | 13519206 | 0.853 | 0.007 | 0.258 | 0.079 | 0.261 | | 0.120 |
| 113 | 131847040 | 0.874 | 0.005 | 0.334 | 0.048 | 0.262 | | 0.120 |
| 114 | 14015977 | 0.848 | 0.007 | 0.411 | 0.027 | 0.287 | | 0.098 |
| 115 | 71314876 | 0.881 | 0.005 | 0.284 | 0.066 | 0.207 | | 0.187 |
